# Layered rare-earth hydroxides as multi-modal medical imaging probes: particle size optimisation and compositional exploration†

**DOI:** 10.1039/d4dt00371c

**Published:** 2024-04-19

**Authors:** Margarita Strimaite, Connor J. R. Wells, Timothy J. Prior, Daniel J. Stuckey, Jack A. Wells, Gemma-Louise Davies, Gareth R. Williams

**Affiliations:** a UCL School of Pharmacy, University College London 29-39 Brunswick Square London WC1N 1AX UK g.williams@ucl.ac.uk; b UCL Centre for Advanced Biomedical Imaging, University College London 72 Huntley Street London WC1E 6DD UK; c Department of Chemistry, University College London 20 Gordon Street London WC1H 0AJ UK g.davies.7@bham.ac.uk; d Chemistry, School of Natural Sciences, University of Hull Kingston Upon Hull HU6 7RX UK; e School of Chemistry University of Birmingham Edgbaston Birmingham B15 2TT UK

## Abstract

Recently, layered rare-earth hydroxides (LRHs) have received growing attention in the field of theranostics. We have previously reported the hydrothermal synthesis of layered terbium hydroxide (LTbH), which exhibited high biocompatibility, reversible uptake of a range of model drugs, and release-sensitive phosphorescence. Despite these favourable properties, LTbH particles produced by the reported method suffered from poor size-uniformity (670 ± 564 nm), and are thus not suitable for therapeutic applications. To ameliorate this issue, we first derive an optimised hydrothermal synthesis method to generate LTbH particles with a high degree of homogeneity and reproducibility, within a size range appropriate for *in vivo* applications (152 ± 59 nm, *n* = 6). Subsequently, we apply this optimised method to synthesise a selected range of LRH materials (R = Pr, Nd, Gd, Dy, Er, Yb), four of which produced particles with an average size under 200 nm (Pr, Nd, Gd, and Dy) without the need for further optimisation. Finally, we incorporate Gd and Tb into LRHs in varying molar ratios (1 : 3, 1 : 1, and 3 : 1) and assess the combined magnetic relaxivity and phosphorescence properties of the resultant LRH materials. The lead formulation, LGd_1.41_Tb_0.59_H, was demonstrated to significantly shorten the *T*_2_ relaxation time of water (*r*_2_ = 52.06 mM^−1^ s^−1^), in addition to exhibiting a strong phosphorescence signal (over twice that of the other LRH formulations, including previously reported LTbH), therefore holding great promise as a potential multi-modal medical imaging probe.

## Abstract 2

A detailed study into the synthesis and functional properties of layered rare-earth hydroxides (LRHs) is reported. It is possible to obtain precise control of particle size, and combining Tb and Gd in the LRH allows multi-modal imaging.

## Graphical abstract

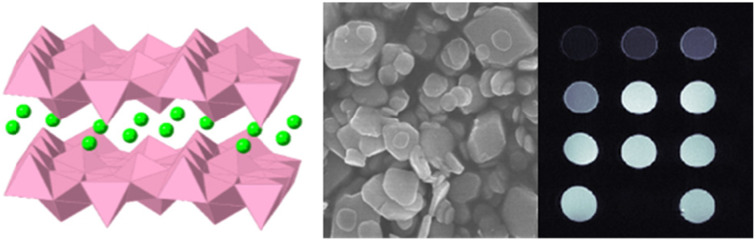

## Introduction

Layered rare-earth hydroxides (LRHs) have the general formula [R_2_(OH)_(6−*m*)_](A_(*m*/*n*)_^*n*−^)·*y*H_2_O, where R is a rare-earth metal ion, and A is an intercalated counterion. They are structurally similar to the more prominent class of materials known as the layered double hydroxides (LDHs), which are based around divalent/trivalent metal ions (commonly Zn^2+^, Mg^2+^, Al^3+^) rather than rare-earth ions. Owing chiefly to their anion-exchange ability and low cost of production, the LDH class of materials have enjoyed substantial attention across a range of applications including pollutant sequestration/remediation,^2,3^ catalysis,^4^ electrode materials,^5^ and sensing,^6^ to name a few.

On the other hand, the anion-exchange ability coupled with the presence of rare-earth cations within LRHs lend themselves particularly well to theranostic applications. The part-filled 4f orbitals of rare-earth ions give rise to a rich range of emissions which can be exploited for photoluminescence imaging, and, particularly in the case of Gd^3+^, exploitable magnetic properties which form the basis for magnetic resonance imaging contrast. The positively charged layered structure of LRHs enables them to reversibly uptake anionic guest species. These guest moieties may be selected to exert a therapeutic effect, as well as to modify the overall contrast properties of the parent LRH material. Moreover, LRH materials have previously been shown to be stable at physiological pH^1^ and well-tolerated in cytotoxicity studies^7,8^ (though *in vivo* applications of LRHs are thus far extremely limited in scope, and long-term toxicity is not yet explored). As such, LRHs are attracting increasing interest in the field of theranostics.

To date, many LRH materials have been successfully synthesised, with a wide range of parent compositions and guest species such as amino acids and drug anions.^9–12^ LRH theranostic platforms based on Gd and Tb have been reported previously by our group;^1,13^ despite favourable overall performance, the reported hydrothermal synthesis protocols produced inhomogeneous mixtures of nano- and microparticles, which without further optimisation are not suitable for translation towards the clinic. The inhomogeneity of previously synthesised LRH particles is problematic, as both size and geometry of particulate formulations affect their performance *in vivo*. Injectable formulations must be able to avoid rapid elimination by the body's biological mechanisms; there is evidence in the literature that larger particles (*ca.* 400 nm upwards) are more susceptible to phagocytic uptake than smaller particles (200 nm and below).^14^ Moreover, for cancer therapy applications, particle size should be appropriate to preferentially accumulate in the leaky tumour vasculature (which may have intracellular gaps larger than 200 nm)^15^ and not healthy endothelium (with intracellular gaps around 12–150 nm).^16,17^ Therefore, for purposes of particle size-optimisation in this work, average particle size between 150–200 nm was targeted.

The parameters of reaction temperature, reaction time, and reactor fill volume during hydrothermal synthesis were chosen to systematically investigate in terms of their influence on resultant LTbH particle morphology. Synthesis temperature affects the viscosity, density, and relative permittivity of water, and therefore the kinetics of product formation as well as solubility of reactants/products. The length of reaction time contributes to selectivity towards the thermodynamic product, and determines the resultant particle size.^18^ Moreover, longer aging times are associated with higher degrees of crystallinity, as has been demonstrated for structurally similar layered double hydroxide (LDH) systems.^19^ The internal system pressure during hydrothermal synthesis is determined by the degree of fill of the reaction chamber as well as the reaction temperature. While the mechanism of formation of LRHs (and LDHs) is not well-understood at present, there is some evidence to imply it may be an oriented attachment process (whereby building blocks of LRH nucleate instantly and subsequently assemble into a 3D structure),^20^ and similar oriented attachment processes have been reported to be influenced by the pressure during synthesis.^21^

The metal composition of LRH materials can be used to modulate their contrast ability. For instance, a recently reported mixed-metal (Y/Tb/Gd) LRH system exhibits magnetic resonance imaging contrast (from Gd^3+^) and green luminescence (from Tb^3+^),^7^ and a Gd/Eu LRH shows magnetic resonance imaging contast and red luminescence from Eu^3+^.^11^ There are also reports of Gd/Tb/Eu LRH and Tb/Y LRH giving colour tuneable luminescence,^22,23^ and a Gd-doped LTbH-NO_3_ system showing vastly enhanced luminescence arising from the synergistic interaction of Gd^3+^ (as well as an intercalated organic sensitiser) and Tb^3+^.^24^ The present study additionally explores the production of several mixed Gd/Tb LRH systems with varying Gd : Tb ratios using the optimised hydrothermal synthesis procedure. These systems are used to investigate how the synergistic interaction between Gd^3+^ and Tb^3+^ centres varies as a factor of composition, and how it influences resultant magnetic relaxivity and luminescence behaviours.

This work first explores whether it is feasible to produce homogeneous populations of LTbH, with an average particle size of *ca.* 150–200 nm, by systematically varying hydrothermal reaction conditions. Secondly, the optimised synthetic method is applied to generate LRHs based on a selection of other rare-earth elements. Lastly, LRHs combining both Tb^3+^ and Gd^3+^ in varying ratios are made to investigate their combined imaging contrast properties. We hypothesized that the presence of Gd would enhance the phosphorescence emanating from Tb^3+^, similar to previous reports.^24^

## Methods

### Materials

Terbium, dysprosium, erbium, and ytterbium chloride hexahydrate salts were purchased from Sigma Aldrich. Praseodymium and gadolinium chloride hexahydrate salts were purchased from Alfa Aesar, and neodymium chloride hexahydrate from Chem Cruz. All water used was deionised, and ethanol was of analytical grade.

### Hydrothermal synthesis of LTbH-Cl with systematically varied parameters

Reaction parameters were determined using the JMP Pro 14 software and are summarised in Table S1 (ESI†). An aqueous solution of TbCl_3_·6H_2_O (0.4 M, either 7.5 ml or 13.5 ml) was added dropwise to a stirring aqueous solution of NaCl/NaOH (1.4 M and 2.1 M respectively, either 2.5 ml or 4.5 ml), such that the total volume was either 10 ml or 18 ml. The resultant suspension was stirred for 10 min and transferred to a Teflon lined hydrothermal reactor (23 ml). The reactor was heated to the desired temperature (90, 100, 140, or 200 °C) for the required length of time (4, 8, 10, or 24 h), at a heating and cooling rate of 10 °C min^−1^, after which the precipitate was collected by centrifugation. The crude product was washed with water (2 × 30 ml) and ethanol (2 × 30 ml), and subsequently dried at 60 °C for 24 h. The product cake was briefly (<1 min) ground using a pestle and mortar to produce a powder.

### Hydrothermal synthesis of LRH-Cl (R = Pr, Nd, Gd, Dy, Er, Yb)

An aqueous solution of RCl_3_·6H_2_O (0.4 M, 13.5 ml, R = Pr, Nd, Gd, Dy, Er, Yb) was added dropwise to a stirring aqueous solution of NaCl/NaOH (1.4 M and 2.1 M respectively, 4.5 ml), such that the total volume was 18 ml. The resultant suspension was stirred for 10 min and transferred to a Teflon lined hydrothermal reactor (23 ml). The reactor was heated to 90 °C (150 °C for LYbH) for 4 h, at a heating and cooling rate of 10 °C min^−1^, after which the precipitate was collected by centrifugation. The product was washed with water (2 × 30 ml) and ethanol (2 × 30 ml), and subsequently dried at 60 °C for 24 h.

### Hydrothermal synthesis of Gd/Tb LRH-Cl

A solution of TbCl_3_·6H_2_O (*X* M, *X* = 0.3, 0.2, or 0.1) and GdCl_3_·6H_2_O (0.4 – *X* M) in water (13.5 ml) was added dropwise to a stirring aqueous solution of NaCl/NaOH (1.4 M and 2.1 M respectively, 4.5 ml), such that the total volume was 18 ml. The resultant suspension was stirred for 10 min and transferred to a Teflon lined hydrothermal reactor (23 ml). The reactor was heated to 90 °C for 4 h, at a heating and cooling rate of 10 °C min^−1^, after which the white precipitate was collected by centrifugation. The product was washed with water (2 × 30 ml) and ethanol (2 × 30 ml), and subsequently dried at 60 °C for 24 h.

## Characterisation

### Powder X-ray diffraction (PXRD)

X-ray diffraction analysis was performed using a Rigaku Miniflex 600 diffractometer with Cu Kα radiation (*λ* = 1.5418 Å). Patterns were collected over the 2*θ* range 3–60°, at 40 kV and 15 mA.

Further X-ray diffraction data were collected in transmission mode using finely ground sample powders sandwiched between two cellulose acetate foils. A Stoe StadiP X-ray diffractometer, operating with Mo Kα_1_ radiation (*λ* = 0.7093 Å) in the 2*θ* range from 2 to 40° with 0.5° detector steps every 5 s (giving a step size of 0.015°) was used. The sample was rotated during the data collection.

The raw data were fitted using the Rietveld method,^25^ within the GSAS-II suite of programmes.^26^ In each case, the initial model employed was the structure of layered ytterbium hydroxychloride, LYbH-Cl (ICSD no. 419745).^27^ The data were fitted using standard procedures, and the background was fitted with a 6-term shifted Chebyschev polynomial. The experimental peak shape was fitted with a pseudo-Voigt function. Unit cell parameters, a single sample-height correction, and the atomic positions for the heavy atoms (Gd, Tb, and Cl) were refined. The small positive isotropic displacement parameters obtained from the known structure were retained. The fitting of the observed data was slightly complicated by the asymmetry of the strong low angle peak around 5°.

### Scanning electron microscopy (SEM)

Samples were sputter coated with gold and imaged using a JEOL JSM-6701F field emission scanning electron microscope. Particle size analysis on the resulting images was performed manually using the ImageJ 1.52a software, and the results presented as mean ± standard deviation. All particles were measured along their longest dimension. The number of particles measured per sample is indicated in Table S2.† Electron dispersive X-ray spectroscopy (EDXS) was performed using the same microscope, with the aid of an INCAx-Act detector (Oxford Instruments).

### Dynamic light scattering (DLS)

The hydrodynamic sizes of the LTbH particles were analysed using a Zetasizer Ultra instrument and ZS Xplorer software (Malvern Panalytical). Suspensions of the particles (0.1 mg ml^−1^) were prepared by re-dispersing the samples in water by ultrasonication (5 min). Disposable folded capillary zeta cells (DTS1070, Malvern Panalytical) were used. Each suspension was measured in triplicate, and the results are given as mean ± standard deviation.

### Elemental microanalysis

Carbon/hydrogen/nitrogen content analysis was performed using flash combustion on a Carlo Erba CE1108 elemental analyser.

### Thermogravimetric analysis (TGA)

Thermogravimetric analysis was performed using a TA Instruments Discovery TGA instrument controlled by the TA Trios software. The samples were heated from 40–900 °C at a rate of 20 °C min^−1^, under nitrogen gas at a flow rate of 25 ml min^−1^.

### Cytotoxicity assay

To assess the cytotoxicity of the formulations, human colorectal adenocarcinoma cells (Caco-2) and human embryonic kidney cells (HEK 293) were used. Caco-2 cells were cultured at 37 °C under 5% CO_2_, in high glucose Dulbecco's modified Eagle's medium (DMEM-HG, 435 ml, Sigma Aldrich) supplemented with the following solutions: penicillin–streptomycin (5 ml, Life Technologies), l-glutamine (5 ml, Life Technologies), non-essential amino acid solution (5 ml, Life Technologies), and foetal bovine serum (50 ml, Gibco). HEK-293 cells were cultured at 37 °C under 5% CO_2_, in Dulbecco's modified Eagle's medium (DMEM, 440 ml, Sigma Aldrich) supplemented with the following solutions: penicillin–streptomycin (5 ml, Life Technologies), l-glutamine (5 ml, Life Technologies), and foetal bovine serum (50 ml, Gibco). The cell cultures were passaged at 50–75% confluence by treatment with trypsin solution (0.25% in EDTA, 2 ml, Sigma Aldrich). For cell viability experiments, the passage numbers used were 73–76 (Caco-2) and 9–12 (HEK 293).

Cell viability assessments were conducted using the CellTiter-Glo™ assay, in white 96-well plates (Corning). Cell suspension was prepared at a concentration of 5.6 × 10^4^ cells per ml and deposited into the wells of the plate (180 μl each). LRH suspensions in culture medium (10 mg ml^−1^) were prepared, and either 5 or 10 μl of these suspensions were added directly to the cells. Samples used in this series of experiments were LRH-Cl (R = Pr, Nd, Gd, Tb, Dy, Er, and Gd/Tb).

For background luminescence measurements, wells were prepared using only cell culture medium in place of cell suspension. For control cell luminescence measurements, wells were prepared by using cell culture medium in place of LRH suspensions. The plates were then incubated at 37 °C for 24 h. After incubation, CellTiter-Glo™ 2.0 assay reagent (100 μl, Promega) was added to each well, and left to react at room temperature for 30 min. The luminescence of the plates was read over the range 250–850 nm using a SpectraMax M2e microplate reader (Molecular Devices). Cell viability for each well was calculated according to eqn (1):1
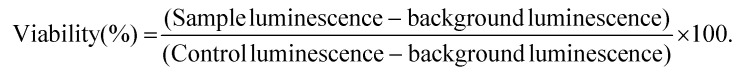


Three independent experiments were performed, each with treatment conditions in triplicate, and results presented as mean ± standard deviation.

### Time-resolved fluorescence (TRF)

Photoluminescence measurements were carried out in black 96-well plates using a SpectraMax M2e microplate reader (excitation = 250 nm, emission = 545 nm). LRH samples (*ca.* 30 mg) were suspended in water (10 ml), and 150 μl of these suspensions was transferred to a 96-well plate. Time-resolved fluorescence measurements were taken after a delay of 50 μs and measured up to 1450 μs (in increments of 250 μs).

### Inductively coupled plasma optical emission spectroscopy (ICP-OES)

Inductively coupled plasma optical emission spectroscopy (ICP-OES) was conducted on a Varian 720-ES ICP-OES instrument. Stock suspensions of samples were prepared at a concentration of *ca.* 30 mg in 10 ml distilled water. 1 ml of these stock suspensions was then acid digested using hot nitric acid and diluted with distilled water to 10 ml. The samples were then measured against a standard containing either Gd^3+^ or Tb^3+^ (100 mg L^−1^ in 5% HNO_3_ and 1000 mg L^−1^ in 2% HNO_3_ respectively). The standard was diluted to aliquots of 2, 4, 6, and 8 ppm to make a calibration curve.

### Magnetic resonance imaging (MRI)

Stock suspensions of LGd_1.41_Tb_0.59_H were prepared at a concentration of 10 mg in 10 ml distilled water. Serial dilutions of these stock suspensions were then performed to obtain suspensions in the concentration range 0.035–0.9 mg ml^−1^ (equivalent to final Ln^3+^ concentrations in the range 0.08–1.72 mM). Polypropylene well plates were cut into sections (3 × 4 wells, 300 μl well volume) to prepare imaging phantoms. Sample suspensions (300 μl) were pipetted into each well, and sealed with a polypropylene cap. The dilution series was replicated three separate times.

Images were obtained using a preclinical MRI system (Agilent) operating at 400 MHz, 9.4 T, and 18 °C with a 38 mm quadrature driven birdcage RF coil (Rapid Biomedical). For *T*_1_-weighted images, an inversion recovery fast spin echo acquisition was used (repetition time (TR) = 10 s, echo train length (ETL) = 4, matrix = 128 × 128, inversion time (TI) = 100, 185, 343, 636, 1180, 2200, 4000, and 7500 ms). For *T*_2_-weighted images, a multi-echo sequence was employed (TR = 4 s, echo time (TE) = *ca.* 8 ms, number of echoes (NE) = 32, matrix = 128 × 128). *T*_1_ and *T*_2_ values were subsequently calculated for each region of interest using MatLab scripts.

The measured relaxation times were then used to calculate corresponding relaxation rates, 1/*T*_*x*_ (*x* = 1 or 2, as appropriate). The observed relaxation rate can be expressed according to eqn (2), where *r*_*x*_ is the relaxivity, and [Ln^3+^] is the concentration of Ln^3+^ ions (in mM), and (1/*T*_*x*_)_*w*_ is the relaxation rate of pure water.2
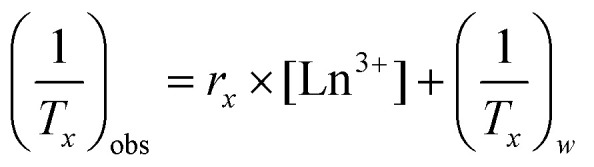


The Ln^3+^ concentrations for each suspension were calculated from the experimentally derived formula (Table 4, LGd_1.41_Tb_0.59_H). The formula was entered into a molecular weight calculation software (Molecular Weight Calculator 6.5, Matthew Monroe) to obtain the average formula mass (463.60 g mol^−1^). The formula mass was then used to convert suspension concentrations from units of mg ml^−1^ to mM, and the latter were then doubled to account for the presence of two Ln^3+^ ions per formula unit.

The relaxivities (*r*_1_ and *r*_2_ (s^−1^)) for each LRH formulation were then derived from the fitted gradients of the plots of relaxation rate (1/*T*_*x*_) against [Ln^3+^] (mM) for each sample.

### Statistical analysis

One-way analysis of variance (ANOVA) was used to determine the statistical significance (at the *p* = 0.05 level) in the difference of means for particle size measurements. A Fisher's LSD *post-hoc* test was used to determine which sample means differed significantly. The mean particle size values for each set of experimental parameters were input into JMP Pro 14 software for model fitting and effect screening.

## Results and discussion

### Particle size optimisation

For initial synthesis optimisation experiments, the temperature was set to 100 °C and 200 °C (50 °C above and below the previously reported method),^1^ with incubation times of 10 and 24 h (compared to 15 h originally). The fill volumes chosen were approximately half the capacity of the reactor (10 ml) and full capacity (18 ml), whilst leaving some room for air in order to prevent vessel leakage. These parameters and their combinations are summarised in Table S1† (samples 100-*X-Y* and 200-*X-Y*, where *X* = incubation time, and *Y* = fill volume).

Analysis of XRD patterns (representative patterns shown in Fig. S1†) confirmed that all samples produced at 100 °C were LTbH, whereas those produced at 200 °C were a different phase. There are no reflections present in the 200 °C samples which correspond to LTbH, showing that a full phase conversion has occurred. The powder pattern is consistent with an orthorhombic form of Tb(OH)_2_Cl (Fig. S2†), based on data previously reported for Ln(OH)_2_Cl.^28^

As a phase transition occurs somewhere between 150–200 °C, and particles generated at 150 °C are inhomogeneous, further experiments were carried out at reduced temperatures of 90 °C and 140 °C. In addition, the incubation time was shortened to 4 h and 8 h, both to increase time efficiency, and to potentially further reduce particle size. XRD data (Fig. S1†) confirmed that the samples produced at 90 °C and 140 °C were also LTbH.

**Fig. 1:**
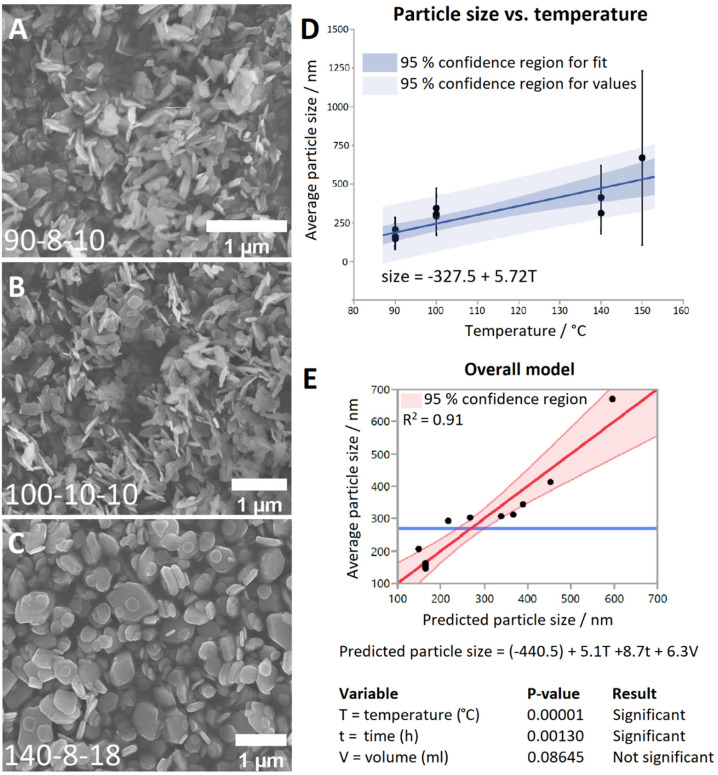
SEM images of LTbH synthesised at (A) 90 °C; (B) 100 °C; (C) 140 °C; (D) plot showing the relationship between synthetic temperature and mean particle size; (E) plot showing the complete model (which includes *T*, *t*, and *V* as variables) fitted to the data, and the statistical impact of each variable on the model. Plots D and E were generated using JMP Pro 14 software.

Representative SEM images from each temperature group are shown in Fig. 1A–C. It can be seen that at 90 °C the particles display a predominantly rod-shaped geometry, whereas at 140 °C the particles are almost exclusively platelet shaped. Samples synthesised at 100 °C showed a preference for the rod morphology, though to a lesser degree than the 90 °C samples. By contrast, samples of the material generated at 200 °C are composed of needle-shaped crystals which are several orders of magnitude larger than LTbH (Fig. S3†).

The mean particle sizes measured for each LTbH sample are summarised in Table S2,† and statistical analysis results are presented in Table S3.† There appears to be a strong correlation between synthesis temperature and resultant particle size measured by SEM (Fig. 1D), with a contributing effect from reaction time, and no significant influence from fill volume (Fig. 1E). All LTbH samples in the present study had a mean particle size significantly smaller than that of samples synthesised according to the previously reported method (at 150 °C).^1^ Furthermore, particles in samples 90-8-10 and 90-4-18-1 (90 °C) were significantly smaller than those from samples synthesised at 100 °C and 140 °C, and particles from sample 140-8-18 (140 °C, with longer incubation time and higher fill volume) were statistically the largest. There are also statistical differences within each temperature group, which are explained by secondary effects from the differences in reaction time.

The experimental parameters producing particles with an average size smaller than 200 nm (90 °C, 4-hour incubation, and 18 ml fill volume) were selected as optimal for cancer theranostics. To verify that this method can consistently produce particles with a predictable size distribution and an average size of less than 200 nm, the experiment was repeated five additional times (Table S2,† samples 90-4-18-*Z*, where *Z* = replicate number). Particle size data measured by SEM are summarised in Table 1, and statistical analysis results are presented in Table S3.† XRD data (Fig. S4†) verified the material was LTbH in all cases. Statistical analysis showed that the mean particle sizes (by SEM) of the replicates (Table S3,† samples 90-4-18-Z, *Z* = 1–6) are not statistically different. Therefore, the optimal method shows good reproducibility in terms of mean particle size and size distribution, compared to other hydrothermally synthesised LRH chlorides, such as LGdH-Cl (rods and platelets, 100 nm–2 μm)^13^ and LYH-Cl (platelets, 200–500 nm).^29^ The reverse microemulsion method has previously been applied to generate more monodisperse LGdH-Cl particles (200–300 nm),^30^ though the particle morphology in that case was less regular than reported here, and the use of surfactants introduced additional components into the system (which are potentially undesirable in pharmaceutical formulations).

**Table 1:** Summary of particle size data from SEM and DLS for replicates of LTbH synthesised at 90 °C

Sample name	Size by SEM (nm)	Particles measured	Hydrodynamic diameter (nm)	Polydispersity index
90-4-18-1	162 ± 85	460	199 ± 3	0.15 ± 0.04
90-4-18-2	151 ± 56	472	196 ± 2	0.18 ± 0.02
90-4-18-3	159 ± 52	515	199 ± 2	0.17 ± 0.03
90-4-18-4	146 ± 49	398	194 ± 6	0.15 ± 0.05
90-4-18-5	147 ± 47	849	194 ± 1	0.18 ± 0.04
90-4-18-6	152 ± 61	840	201 ± 2	0.20 ± 0.02
Overall	152 ± 59	3534	197 ± 4	0.17 ± 0.04

The hydrodynamic diameter of the size-optimised particles, measured by DLS (Table 1), is approximately 50 nm larger than the size measured from SEM images, which is to be expected as this diameter includes water molecules associated with the particles being measured. All samples have low polydispersity, though in some cases a small secondary population was observed around 4–6 μm (Fig. S5†). This is believed to be a consequence of preparing suspensions from dried LRH materials, as a result of aggregated particles which were not sufficiently broken up by grinding and sonication. This is not expected to present a problem for *in vivo* applications as the product does not need to be dried after synthesis.

### Application of optimised synthetic method to other LRHs (R = Pr, Nd, Gd, Dy, Er, Yb)

**Fig. 2:**
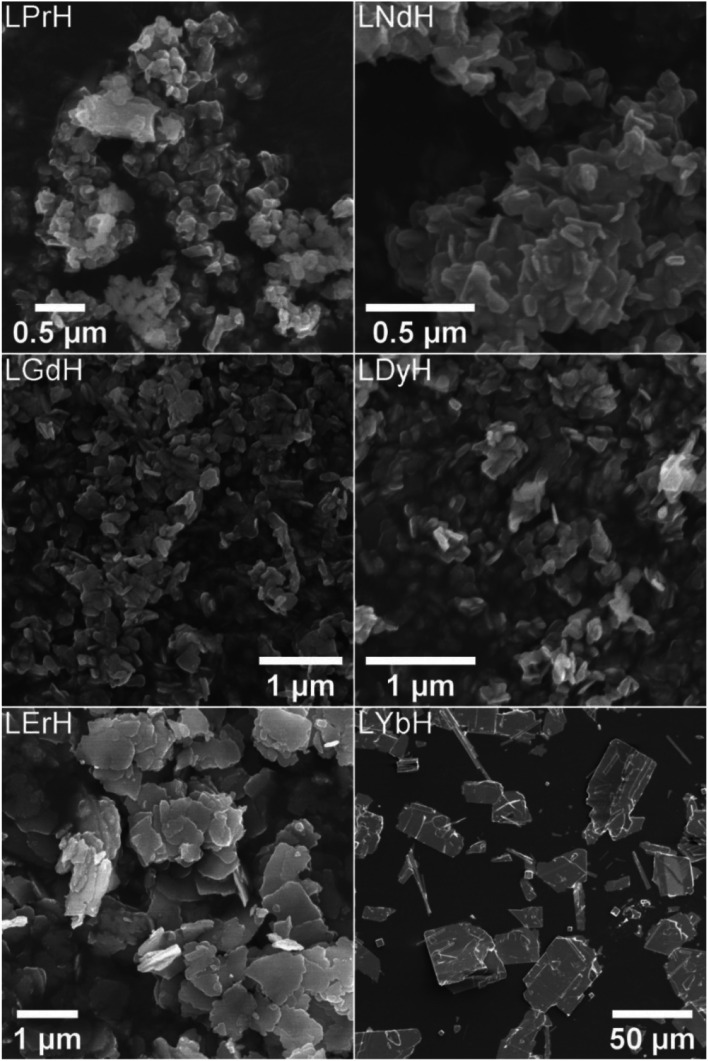
SEM images of the LRH materials (R = Pr, Nd, Gd, Dy, Er, Yb) synthesised at 90 °C (150 °C in the case of LYbH).

The optimal synthesis developed for LTbH was applied to other Ln systems. SEM images of the synthesised LRH materials are shown in Fig. 2, and average particle sizes in Table 2. In general, there appears to be a loose inverse relationship between decreasing Ln^3+^ ion size and particle size. The literature, however, presents a somewhat conflicting picture of how lanthanide ion size relates to LRH particle dimensions. The seminal work which first reported the hydrothermal synthesis of LRH materials based on yttrium and the lanthanides Gd–Lu^31^ did not note differences in resultant particle size. In another study, using the homogeneous precipitation method, the lateral particle size of LRHs was seen to decrease across the period (from Sm–Tm).^32^ Conversely, in a study on the effects of Ln^3+^ doping on the resultant size of layered yttrium hydroxide (synthesised hydrothermally), it was found that inclusion of lighter lanthanide dopants led to smaller overall particle size.^33^

**Table 2:** Summary of mean particle size and number of particles measured for various LRH materials (R = Pr, Nd, Gd, Tb, Dy, Er, Yb) synthesised at 90 °C (*: in the case of LYbH synthesis was carried out at 150 °C). Approximately 80–100 individual particles measured per sample

Sample	Size from SEM	Sample	Size from SEM
LPrH	138 ± 109 nm	LDyH	169 ± 56 nm
LNdH	102 ± 33 nm	LErH	442 ± 246 nm
LGdH	171 ± 67 nm	LYbH*	20 ± 14 μm

LRHs based on Nd, Gd, and Dy exhibit very similar rod and plate shaped particles to the analogous LTbH material (sample 90-4-18, Fig. 1A). Particles in the LPrH sample were highly aggregated. It was therefore challenging to resolve individual particles in the images, though they appear to be of similar dimensions to LRHs with R = Nd–Dy at 100–200 nm. LErH is seen to form thin, plate shaped particles with serrated edges, which bear little resemblance to those of any other LRH materials generated, including the plate shaped LTbH particles synthesized at 140 °C (Fig. 1C). The average size of LErH particles was found to be 442 ± 246 nm, significantly larger than the other LRH materials generated using the optimised protocol at 90 °C.

Crystalline LYbH could not be prepared at 90 °C, instead forming a colloidal dispersion. The synthetic temperature was therefore increased to 150 °C. The particles of LYbH produced are several orders of magnitude larger than the other LRH samples. The majority of LYbH particles are plate shaped, but some crystals are seen which exhibit unidirectional growth, which may be due to the presence of a secondary phase.

**Fig. 3:**
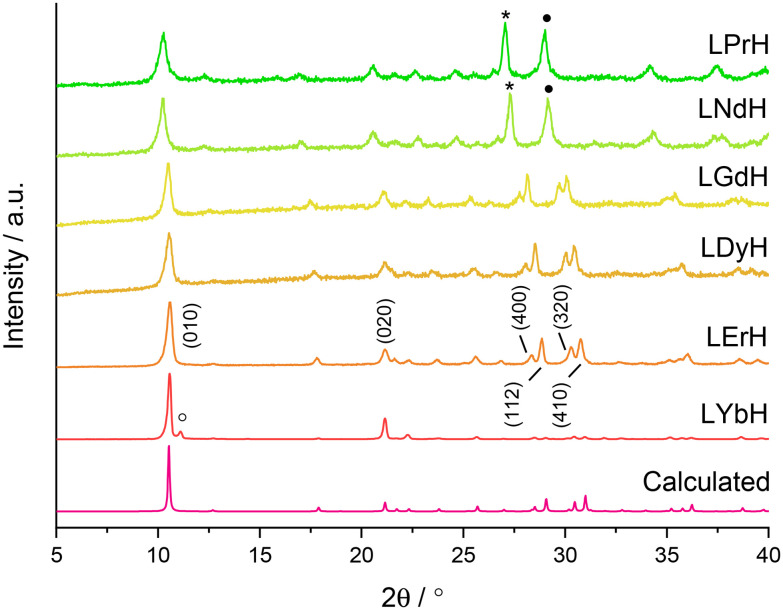
XRD patterns for the LRH materials. (R = Pr, Nd, Gd, Dy, Er, Yb). *: (202) and (400) plane reflections are overlapping, •: (212) and (410) plane reflections are overlapping, °: peak arising from secondary phase of LYbH. Calculated pattern based on LYbH (ICSD collection code 419745).

XRD patterns of the LRH materials (R = Pr, Nd, Gd, Dy, Er, Yb) are shown in Fig. 3. All patterns exhibit the expected (010) reflection just above 2*θ* = 10° and can be indexed in the orthorhombic space group *Pca*2_1_ (refined unit cell parameters are summarised in Table S4†). Interlayer spacing is seen to decrease slightly with decreasing ionic size as previously reported.^34^ All samples also exhibit prominent reflections arising from the (400), (112), (320), and (410) planes, with the exception of LYbH, presumably due to preferred orientation effects associated with the large, sheet like crystals. In general, most reflections shift to higher diffraction angles as the lanthanide ion size decreases, which is due to the decrease in Ln–O distances within the layers.^34^

All samples appear to be phase pure, except LYbH in which a small reflection can be seen next to the (010) reflection (Fig. 3). This peak corresponds to a secondary phase, with a smaller interlayer spacing of 7.96 Å, and has been reported previously.^27^ This is also consistent with the observation of two distinct morphologies for LYbH in the SEM images (Fig. 2).

Thermograms of the various LRH materials are given in Fig. S6.† All compounds exhibit the expected triphasic mass loss. The first mass loss event below *ca.* 200 °C is attributed to the loss of interlayer water, and the corresponding mass loss (along with elemental analysis data) is summarised in Table S5.† This corresponds to between 1 and 1.75 molecules of water per formula unit, summarised in Table 3. Overall, there does not appear to be any trend in the hydration level of the LRH materials. It is likely that the interlayer water content is sensitive to drying and storage conditions, causing natural variability between samples.

**Table 3:** Summary of formulae derived from TGA/elemental analysis data. *Data from prior study.^1^

Material	Formula	Material	Formula
LPrH-Cl	Pr_2_(OH)_5_Cl_0.1_(CO_3_)_0.45_·H_2_O	LDyH-Cl	Dy_2_(OH)_5_Cl_0.7_(CO_3_)_0.15_·H_2_O
LNdH-Cl	Nd_2_(OH)_5_Cl_0.1_(CO_3_)_0.45_·1.75H_2_O	LErH-Cl	Er_2_(OH)_5_Cl_0.8_(CO_3_)_0.1_·1.5H_2_O
LGdH-Cl	Gd_2_(OH)_5_Cl_0.7_(CO_3_)_0.15_·1.75H_2_O	LYbH-Cl	Yb_2_(OH)_5_Cl_0.95_(CO_3_)_0.025_·1.5H_2_O
LTbH-Cl*	Tb_2_(OH)_5_Cl_0.9_(CO_3_)_0.05_·1.5H_2_O		

The results of the elemental analysis and water content as determined by TGA (Table S5†) were used to calculate the approximate formulae of the LRH materials (Table 3). The carbon content of LPrH and LNdH indicated the presence a higher-than-expected amount of carbonate anions, making up 90% of the anions within the interlayer gallery. It is not known why this might be the case, but it may be related to differences in charge density of the anions and lanthanide cations. This is supported by the fact that carbon content seemingly decreases with Ln^3+^ ion size – with carbonate ions accounting for only 5% of the interlayer anions in LYbH. No clear trend in carbonate content was reportedly observed for homogeneously precipitated LRHs with R = Nd–Tm,^34^ but previous studies on LDH materials have shown a strong relation between anion selectivity and the charge density of the constituent LDH layers.^35^

**Fig. 4:**
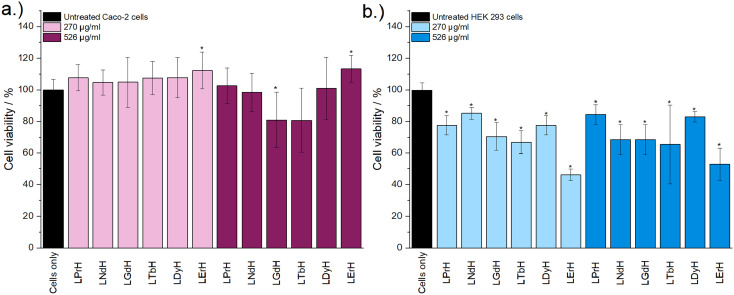
The results of *in vitro* cell viability assays performed with (a) Caco-2 and (b) HEK 293 cell lines and aqueous LRH suspensions at two concentrations (270 μg ml^−1^ and 526 μg ml^−1^). The assay experiment was performed three separate times, each in triplicate. Results are presented as mean ± standard deviation. * Denotes groups with a significantly different mean viability from the control, at the *p* = 0.05 level.

Cytotoxicity of the LRH formulations was evaluated using *in vitro* cell viability experiments on Caco-2 and HEK 293 cell lines (Fig. 4). LYbH was not tested due to its unsuitable particle size. In the Caco-2 cell line experiments, most treatment groups appear to show no statistically significant cytotoxicity at the *p* = 0.05 level. Conversely, for the HEK 293 cell line, a degree of cytotoxicity is seen in all treatment groups at both concentrations (with mean viability of less than 90%). LErH appears to exhibit the most adverse effect on the HEK 293 cells, with *ca.* 50% or less cells surviving after 24 hours, even at the lower testing concentration (270 μg ml^−1^). This effect may be due to particle morphology, as LErH exhibits a mean particle size of 442 ± 246 nm with serrated edges, larger than the Pr/Nd/Gd/Tb/Dy LRHs (mean particle size under 200 nm, generally more smooth rods or plates). Additional factors, such as Ln^3+^ ion identity or differences in LRH solubility may also contribute, though erbium compounds are not known to be particularly toxic relative to the rest of the lanthanides.^36^

To develop a theranostic platform with the most promising combination of features, the lanthanide elements gadolinium and terbium were selected for further study. Terbium exhibits strong luminescence, while gadolinium has a large magnetic moment. SEM images in the current study revealed that both LGdH and LTbH form homogeneous particles of similar size under the optimised synthetic conditions, and their similar ionic size would likely result in high compatibility in a combined crystal system.

### Application of optimised synthetic method to generate mixed Gd/Tb LRH systems

LRH systems containing three different ratios of Gd and Tb were synthesised using feedstock molar ratios of 1 : 3, 1 : 1, and 3 : 1. Inductively coupled plasma optical emission spectroscopy (ICP-OES) was used to verify the Gd/Tb composition of each material (Table 4). ICP-OES, TGA (Fig. S7†), and elemental analysis data (Table 4 and Table S6†) were used to derive the formulae of the three LRH materials (Table 4). The TGA data (Fig. S7†) indicates that all three compounds exhibit the expected triphasic mass loss, as described above for the other LRH formulations. The first mass loss event, corresponding to the loss of interlayer water below *ca.* 200 °C, indicates that all three formulations have very similar water content (approximately 1.6 molecules per formula unit). Elemental mapping (Fig. S8†) indicates a homogenous distribution of Tb and Gd in the LGd_1.41_Tb_0.59_H-Cl system, consistent with formation of a single mixed-metal phase.

**Table 4:** The Gd/Tb fractions of mixed-metal LRH materials (LGd_2−*x*_Tb_*x*_H-Cl), as determined by ICP-IOS (and expected based on concentrations in the synthetic feedstock). Each value is the mean of three separate samples. Formulae derived from ICP-OES/TGA/elemental analysis data

Feedstock ratio (Gd : Tb)	Tb fraction (*x*)	Gd fraction (2 − *x*)	Formula and sample name
3 : 1	1.590 ± 0.003 (1.50)	0.410 ± 0.003 (0.50)	Gd_1.41_Tb_0.59_(OH)_5_Cl_0.78_(CO_3_)_0.11_·1.6H_2_O (LGd_1.41_Tb_0.59_H-Cl)
1 : 1	1.121 ± 0.007 (1.00)	0.879 ± 0.007 (1.00)	Gd_0.88_Tb_1.12_(OH)_5_Cl_0.78_(CO_3_)_0.11_·1.6H_2_O (LGd_0.88_Tb_1.12_H-Cl)
1 : 3	0.591 ± 0.006 (0.50)	1.409 ± 0.006 (1.50)	Gd_0.41_Tb_1.59_(OH)_5_Cl_0.75_(CO_3_)_0.125_·1.6H_2_O (LGd_0.41_Tb_1.59_H-Cl)

In each formulation, the Tb^3+^ content is slightly higher than expected based on the ratio of salts present during synthesis. It is possible that this effect is related to ion size/effective nuclear charge effects, with the slightly smaller, more charge dense Tb^3+^ ions crystallising into the LRH matrix more readily.

**Fig. 5:**
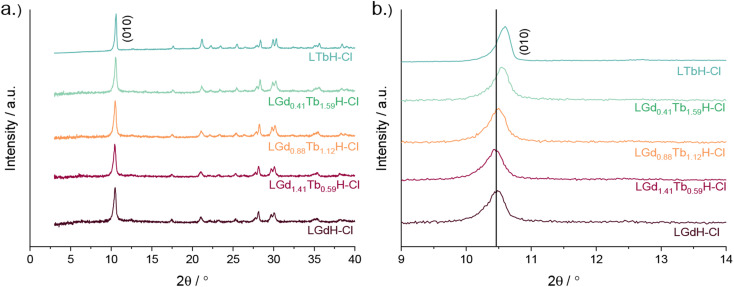
XRD patterns for various compositions of LRH materials (R = Gd, Tb) showing (a) the full patterns and (b) and enlargement of the low-angle region.

XRD patterns of the LRH materials (R = Gd/Tb) with varying composition are shown in Fig. 5. In all cases, the patterns are very similar to each other and to the single-metal LRH materials (LTbH and LGdH). All mixed-metal LRH sample XRD patterns exhibit the expected (010) reflection just above 2*θ* = 10°. The (010) reflection, which corresponds to the interlayer spacing, is seen to shift very slightly to higher diffraction angles with higher Tb^3+^ content. This is consistent with the expectation that the interlayer distance should decrease for heavier lanthanides with a smaller ionic size.^34^

**Fig. 6:**
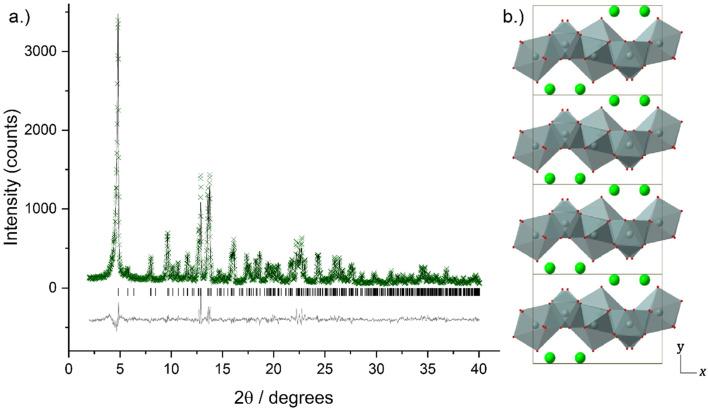
(a) Observed (crosses), calculated (upper line), and difference (lower line) profiles for Rietveld refinement of LGd_1.41_Tb_0.59_H-Cl. Tick marks show the positions of allowed reflections. (b) Crystal structure of LGd_1.41_Tb_0.59_H-Cl viewed down the crystallographic *c*-axis. LnO_*n*_ polyhedra are shown in grey, oxygen atoms positions are marked in red, and inter-layer chloride anions are shown in green.

Higher resolution diffraction patterns of LTbH-Cl, LGdH-Cl, and LGd_1.41_Tb_0.59_H-Cl were collected using a diffractometer with a molybdenum anode for Rietveld refinement based on the previously reported layered ytterbium hydroxychloride (LYbH-Cl)^27^ structure. Inspection of the powder diffraction patterns (Fig. 6, S9 and S10†) revealed that they were broadly similar to each other as well as to LYbH-Cl. Rietveld refinement using the reported LYbH phase as the starting model proceeded smoothly for LGd_1.41_Tb_0.59_H-Cl and LGdH-Cl. A representative Rietveld fit for LGd_1.41_Tb_0.59_H-Cl is shown in Fig. 6a, and confirms the soundness of the structural model. The remaining fits are presented in Fig. S9 and S10,† and the refined unit cell parameters are summarised in Table S7.† Though it was possible to fit LTbH-Cl (Fig. S10†), this phase was much less crystalline than either of the Gd-containing phases. CSD 2328393–2328395 contain the structural data for these crystal structures and are available from the CCDC/FIZ Karlsruhe data access service.

The basic structure prototype for these solids has been reported before,^27^ so the structural description here is brief and focuses on the example of LGd_1.41_Tb_0.59_H-Cl. The structure crystallises in the non-centric space group *Pca*2_1_, with two independent Ln^3+^ ions in the asymmetric unit. These are coordinated by 7 and 8 hydroxide ions respectively, and each is bound to one water molecule. The coordination geometry of Ln(1) is irregular, and that of Ln(2) may be described as a capped square antiprism. The hydroxide ions each coordinate to three Ln^3+^ ions to generate a dense layer of linked polyhedra that extend in the *xz* plane. No hydrogen atoms were located in the original study, and have not been added to the present model, but the orientation of the oxygen atoms strongly suggests that the O–H of the hydroxide groups point into the interlayer region. Water molecules project above and below each layer. Between the layers are located the chloride ions, forming hydrogen bonds to bound water and hydroxide. The structure is shown in Fig. 6b.

There is no evidence in the current diffraction data for ordering of the Tb^3+^ and Gd^3+^ ions within LGd_1.41_Tb_0.59_H-Cl. As expected, the unit cell of LGd_1.41_Tb_0.59_H-Cl contracts relative to LGdH-Cl with the partial replacement of Gd^3+^ by Tb^3+^ due to the well-known lanthanide contraction phenomenon (*V* = 782.32(5) and 791.06(6) Å^3^ respectively, Table S7†).

**Fig. 7:**
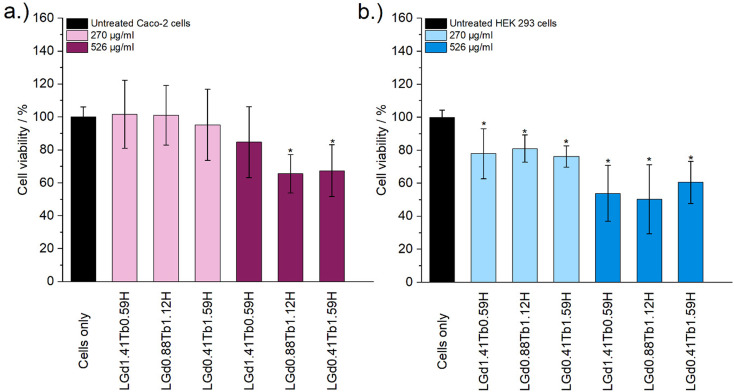
The results of *in vitro* cell viability assays on (a) Caco-2 and (b) HEK 293 cell lines, performed with LRH materials of varying composition at two concentrations (270 μg ml^−1^ and 526 μg ml^−1^). The experiment was performed three separate times, each in triplicate. Results are presented as mean ± standard deviation. * Denotes groups with a significantly different mean viability from the control, at the *p* = 0.05 level.

Cytotoxicity of the mixed Gd/Tb LRH formulations was evaluated using *in vitro* cell viability experiments on Caco-2 and HEK 293 cell lines (Fig. 7). For the Caco-2 cell line, no LRH (R = Gd, Tb) composition shows a statistically significant change in cell viability relative to the untreated control at the lower treatment concentration (270 μg ml^−1^). At the higher concentration of 526 μg ml^−1^, the viability of cells treated with LGd_0.88_Tb_1.12_H-Cl and LGd_0.41_Tb_1.59_H-Cl declines slightly to approximately 70% relative to the untreated control. The marginally higher cell viability after treatment with LGd_1.41_Tb_0.59_H-Cl may be related to the slightly higher Gd content, as has been reported for LGdH-Cl previously.^13^ Overall, LRH composition does not appear significantly affect the viability of Caco-2 cells, but may have some influence at higher treatment concentrations. Some cytotoxicity is seen in all HEK 293 cell treatment groups. It seems that HEK-293 cells are more sensitive to the presence of LRH materials than Caco-2 cells; this is broadly in agreement with cytotoxicity data presented for LTbH-Cl^1^ and LGdH-Cl/LTbH (current study). Additionally, the HEK 293 cell line has been previously demonstrated to be more sensitive than cancerous HeLa cells to the presence of silver nanoparticles,^37^ as well as experiencing mild inhibition (*ca.* 95% viability) in the presence of only 10 μg ml^−1^ of Gd/Tb-containing nanoparticles.^38^

The limited existing studies regarding the cytotoxicity of LRH materials are typically conducted using lower treatment concentrations (50–100 μg ml^−1^).^9,10,39^ Therefore, the present findings of relatively high cell viability (*ca.* > 70%) in most treatment groups across both cell lines for the lower concentration tested (270 μg ml^−1^) provide strong evidence that these materials are well-tolerated. Compared to other Ln-containing systems explored for biomedical applications, for instance Gd-containing quantum dots (*ca.* 70% viability in Caco-2 at 400 μg ml^−1^)^40^ and Gd/Tb containing silica nanoparticles (*ca.* > 75% viability in Caco-2 at 100 μg ml^−1^),^41^ the LRH materials studied in the present work do not appear to exhibit any additional cytotoxicity.

**Fig. 8:**
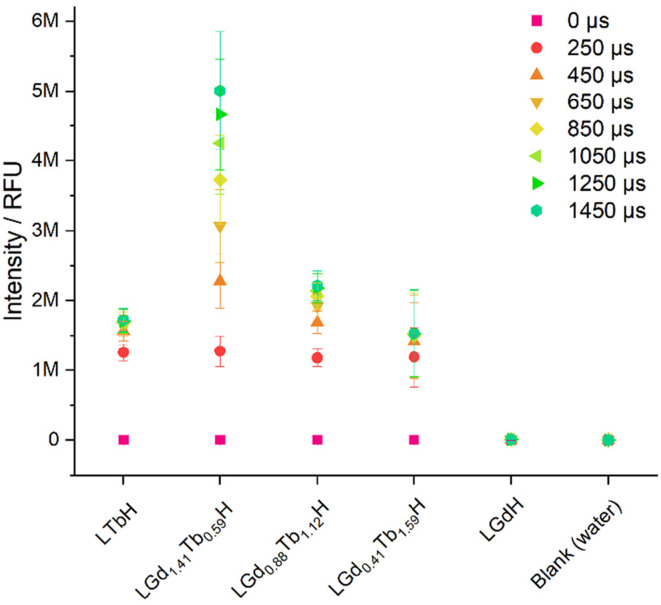
Plot showing the luminescence intensity (ex/em = 250/545 nm) in aqueous suspensions of LRH materials (3 mg ml^−1^) with varying composition, at different integration times (0–1450 μs). Results are presented as mean of three separate samples ± standard deviation.

The photoluminescence response of the various compositions of LRH (R = Gd, Tb) was compared in aqueous suspension (3 mg ml^−1^) by using time-resolved fluorescence (TRF; Fig. 8). By far the highest maximum luminescence intensity reached after 1450 μs is seen in LGd_1.41_Tb_0.59_H-Cl (*ca.* 4–6 M relative fluorescence units). The remaining Gd/Tb LRH samples and LTbH-Cl exhibit comparable luminescence intensity, though the luminescence of LGd_0.88_Tb_1.12_H-Cl is slightly more intense than LTbH-Cl.

Overall, this is likely a combination of two effects – firstly, Tb^3+^–Tb^3+^ quenching effects are minimised with higher Gd^3+^ content (as there is more separation between Tb^3+^ centres on average).^24^ Secondly, additional energy transfer from Gd^3+^ to Tb^3+^ occurs, resulting in a higher population of excited states in the Tb^3+^ centres and thence higher luminescence production, as has been reported in several types of systems containing both Tb^3+^ and Gd^3+^.^24,42^ This also helps to explain the longer time to reach saturation intensity in LGd_1.41_Tb_0.59_H-Cl compared to the other Tb^3+^ containing samples (over 1450 μs *vs.* less than *ca.* 1050 μs), as the additional energy transfer from Gd^3+^–Tb^3+^ is not instantaneous.

**Fig. 9:**
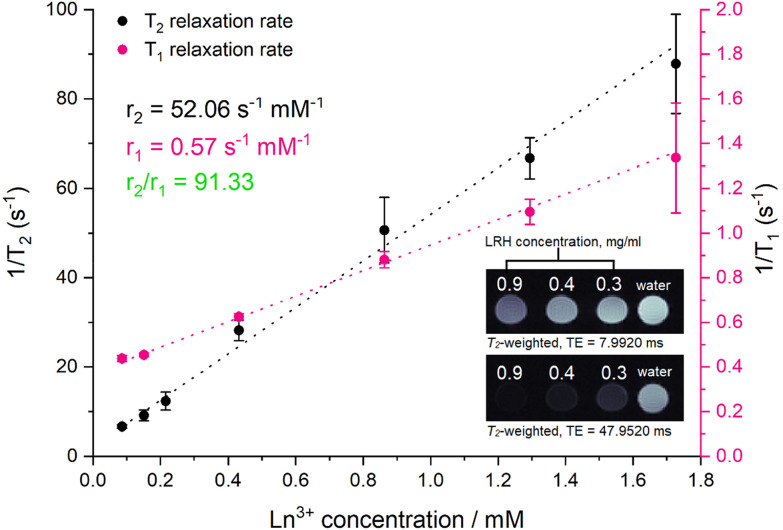
Relaxation rate graphs (*T*_1_ and *T*_2_) of LGd_1.41_Tb_0.59_H constructed from fitted *T*_1_ and *T*_2_ values, which were in turn derived from MR images of a dilution series of LGd_1.41_Tb_0.59_H. Results are presented as mean of three separate samples ± standard deviation. Inset: *T*_2_-weighted images of water and selected aqueous suspensions of LGd_1.41_Tb_0.59_H (0.9. 0.4, 0.3 mg ml^−1^) at different at long and short echo times. Note: images have been cropped and rearranged for illustrative purposes, full images are presented in Fig. S13.†

To investigate the ability of the lead formulation LGd_1.41_Tb_0.59_H-Cl to generate magnetic resonance imaging contrast, serial dilutions in water were prepared and imaged on a 9.4T MRI system (example images at short and long echo times shown in Fig. 9, inset; representative digital, *T*_1_, and *T*_2_-weighted images (at all inversion/echo times measured) shown in Fig. S11–S13†). The fitted *T*_1_ and *T*_2_ values from the images were then used to plot relaxation rate (1/*T*_*x*_) against Ln^3+^ concentration (Fig. 9), and to subsequently calculate the relaxivities for each dilution series from the slope of the graphs. The *r*_1_ relaxivity (0.57 s^−1^ mM^−1^) is low compared to commercially available Gd-based Magnevist and Dotarem contrast agents (4.1 and 3.7 s^−1^ mM^−1^ respectively, in water and measured at 9.4T),^43^ but is in line with previously reported *r*_1_ relaxivity for LGdH (0.51 mM^−1^ s^−1^, in agarose gel at 7T).^13^ This is believed to be a consequence of the layered nature of the LRHs – slow diffusion of water molecules through the interlayer space results in only the surface Gd–OH polyhedra being able to efficiently partake in proton exchange with the surrounding bulk water and contribute to *T*_1_ relaxation. This is analogous to reports on non-layered Gd-phosphate nanoparticles,^44^ where *r*_1_ relaxivities were shown to depend on surface to volume ratio. Additionally, in the present study, *r*_1_ relaxivity is slightly supressed as a result of plotting the relaxation rate against the concentration of both Gd^3+^ and Tb^3+^ (rather than Gd^3+^ alone); both Gd^3+^ and Tb^3+^ affect *T*_2_ relaxation, though only Gd^3+^ contributes significantly to *T*_1_ relaxation.

On the other hand, the *r*_2_ relaxivity (52.06 s^−1^ mM^−1^) of this LRH, while lower than that of conventional negative MRI contrast agents based on superparamagnetic iron oxide (such as Feridex, 105 s^−1^ mM^−1^, in water and measured at 4.7T),^45^ is considerable – and taken together with the lowered *r*_1_ relaxivity, this LRH exhibits strong potential as a negative MRI contrast agent (*r*_2_/*r*_1_ = 91.33). In the *T*_2_-weighted images (Fig. 9 inset) notable reduction in signal relative to pure water is seen at LRH concentrations 0.3–0.9 mg ml^−1^, particularly at longer echo times (full series of images at various echo times shown in Fig. S13†).

This is promising for translation towards the clinic as it indicates that LGd_1.41_Tb_0.59_H could be used to effectively shorten the *T*_2_ relaxation time of protons in tissue and provide negative contrast enhancement. Coupled with the potential for preferential accumulation in tissues which exhibit enlarged intercellular fenestration (for instance, cancer or myocardial infarct tissues) due to the optimised size of the LRH particles described in this work, as well as the strong luminescent signal arising from Tb^3+^ which has been previously shown to be sensitive to drug loading, this platform could provide a valuable pathway for targeting and imaging disease pathologies, as well as quantifying drug release/accumulation at the desired site.

## Conclusions

The particle size and morphology of LTbH was successfully optimised through systematic adjustment of hydrothermal reaction parameters, leading to a significant reduction in both mean particle size and particle size distribution from that previously reported (from 670 ± 564 nm (ref. 1) to 152 ± 59 nm). This new method was subsequently applied to generate a series of LRH materials with alternate composition (R = Pr, Nd, Gd, Dy, Er, Yb). The synthesis was successful for R = Pr, Nd, Gd, and Dy, in all cases yielding particles with mean size under 200 nm, well suited for theranostic applications. Lead elements Gd and Tb were selected based on their favourable imaging properties, and three additional formulations containing these elements in varying ratios were synthesised. The photoluminescence of the various LRH formulations was assessed in aqueous suspension, and it was found that LGd_1.41_Tb_0.59_H-Cl exhibited vastly enhanced fluorescence intensity relative to the other mixed Gd/Tb LRHs, which was attributed to the minimised Tb^3+^–Tb^3+^ quenching and additional energy transfer from Gd^3+^ to Tb^3+^. Magnetic resonance imaging studies revealed that the lead formulation, LGd_1.41_Tb_0.59_H, additionally exhibits promising contrast features, with strong *T*_2_-shortening effect on water (*r*_2_ = 52.06 s^−1^ mM^−1^).

## Conflicts of interest

There are no conflicts to declare.

## Supplementary Material

DT-053-D4DT00371C-s001
