# Supplementary material for: Layered rare-earth hydroxides as multi-modal medical imaging probes: particle size optimisation and compositional exploration
Source: Dalton Trans. 2024 Apr 19;53(19):8429–42. doi: 10.1039/d4dt00371c (PMC11091857; doi:10.1039/d4dt00371c)
Supplement: DT-053-D4DT00371C-s001 [file DT-053-D4DT00371C-s001.pdf]

Electronic Supplementary Material (ESI) for Dalton Transactions.
This journal is © The Royal Society of Chemistry 2024

Layered rare-earth hydroxides as multi-modal medical imaging probes: particle size optimisation and compositional exploration

Margarita Strimaite,[a,b] Connor J. R. Wells,[c] Timothy J. Prior,[d] Daniel J. Stuckey,[b] Jack A. Wells,[b] Gemma L. Davies,[c,e]* Gareth R. Williams[a]*

[a] UCL School of Pharmacy, University College London, 29-39 Brunswick Square, London, WC1N 1AX, UK

[b] UCL Centre for Advanced Biomedical Imaging, University College London, 72 Huntley Street, London, WC1E 6DD, UK

[c] Department of Chemistry, University College London, 20 Gordon Street, London, WC1H 0AJ, UK

[d] Chemistry, School of Natural Sciences, University of Hull, Kingston Upon Hull, HU6 7RX, UK

[e] School of Chemistry, University of Birmingham, Edgbaston, Birmingham, B15 2TT, UK

* authors for correspondence. Email: g.davies.7@bham.ac.uk (GLD); g.williams@ucl.ac.uk (GRW)

Particle size optimisation

Table S1 Summary of reaction parameters generated using the JMP Pro 14 software, and precursor solution volumes for all samples. Sample names are designated as T-X-Y-Z, where T = temperature during synthesis, X = incubation time, and Y = fill volume, and Z = replicate number (if applicable).

Sample name	Temperature (°C)	Incubation time (h)	Total Volume (ml)	Volume of $TbCl_3.6H_2O$ solution (ml)	Volume of NaCl/NaOH solution (ml)
90-8-10	90	8	10	7.5	2.5
90-4-18-1	90	4	18	13.5	4.5
90-4-18-2	90	4	18	13.5	4.5
90-4-18-3	90	4	18	13.5	4.5
90-4-18-4	90	4	18	13.5	4.5
90-4-18-5	90	4	18	13.5	4.5
90-4-18-6	90	4	18	13.5	4.5
100-10-10	100	10	10	7.5	2.5
100-10-18	100	10	18	13.5	4.5
100-24-10	100	24	10	7.5	2.5
100-24-18	100	24	18	13.5	4.5
140-4-10	140	4	10	7.5	2.5
140-8-18	140	8	18	13.5	4.5
200-10-10	200	10	10	7.5	2.5
200-10-18	200	10	18	13.5	4.5
200-24-10	200	24	10	7.5	2.5
200-24-18	200	24	18	13.5	4.5

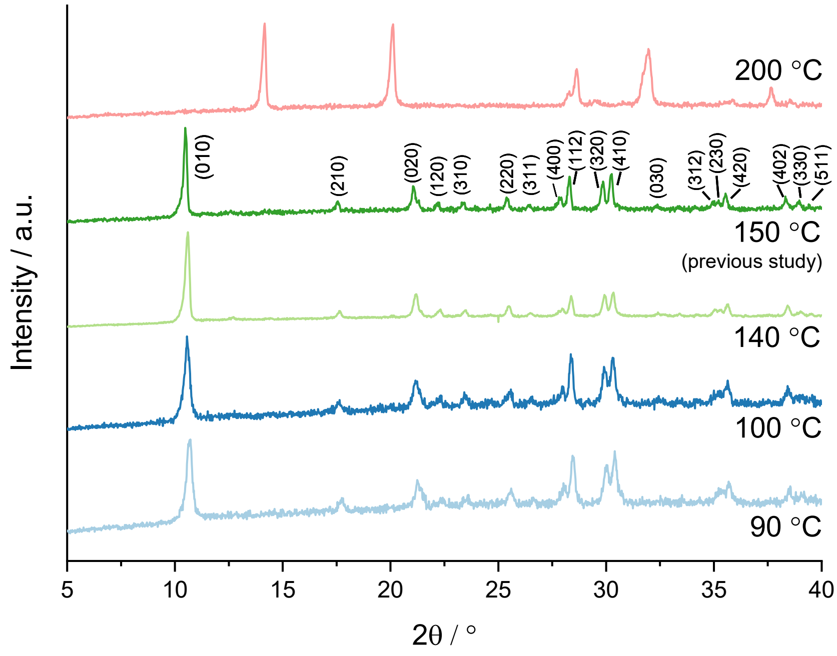

Figure S1 Representative XRD patters for LTbH synthesised according to the previously reported method (at 150 °C),[1] and at 90, 100, and 140 °C; attempted LTbH synthesis at 200 °C yielded an orthorhombic $Tb(OH)_2Cl$ phase. The LTbH system is indexed to the orthorhombic $Pca2_1$ space group.

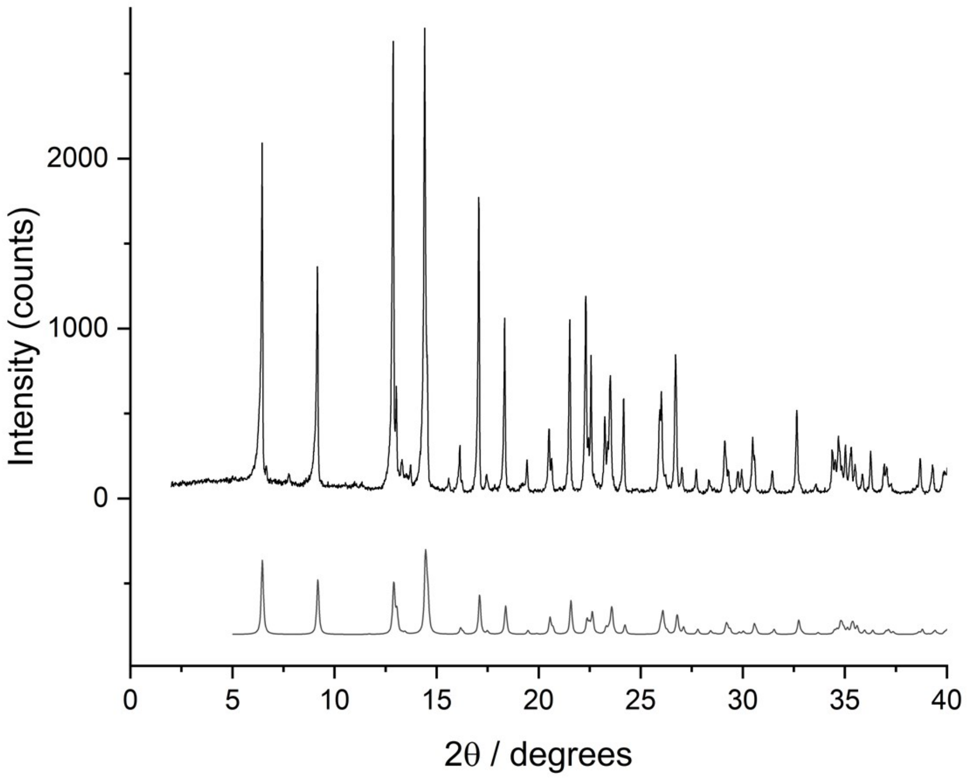

.

Figure S2 Observed X-ray diffraction pattern for phase obtained at 200 °C (above), and simulated pattern for Pnma $Tb(OH)_2Cl$ based on the prototype $Lu(OH)_2Cl$ (below). Observed and simulated patterns are for Mo Kα1 radiation. The phase obtained at 200 °C can be indexed using the primitive orthorhombic cell a = 12.6008(6) Å, b = 3.6638(2) Å, c = 6.2506(3) Å. This is consistent with the Pnma phases $Ln(OH)_2Cl$ (Ln = Tm, Yb, Lu) reported previously.[2] This previous report identified a monoclinic form of $Ln(OH)_2Cl$, but it is clear that a second form exists, orthorhombic $Ln(OH)_2Cl$.

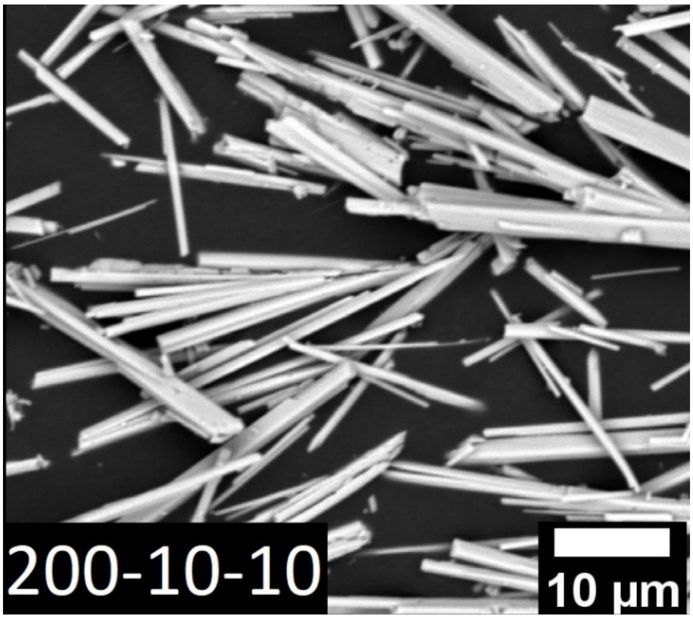

Figure S3 SEM image of orthorhombic $Tb(OH)_2Cl$ phase produced when attempting LTbH-Cl synthesis at 200 °C

Table S2 Summary of average particle sizes from SEM images, hydrodynamic diameters, and polydispersity indexes of materials made under different conditions. Note: none of the above were measured for samples synthesised at 200 °C, as material morphology was not suitable for theranostic applications. Hydrodynamic diameter and polydispersity measurements were conducted only for size-optimised samples.

Sample name	Size by SEM (nm)	Particles measured	Hydrodynamic diameter (nm)	Polydispersity index
90-8-10	206 ± 80	293	n/a	n/a
90-4-18-1	162 ± 85	460	199 ± 3	0.15 ± 0.04
90-4-18-2	151 ± 56	472	196 ± 2	0.18 ± 0.02
90-4-18-3	159 ± 52	515	199 ± 2	0.17 ± 0.03
90-4-18-4	146 ± 49	398	194 ± 6	0.15 ± 0.05
90-4-18-5	147 ± 47	849	194 ± 1	0.18 ± 0.04
90-4-18-6	152 ± 61	840	201 ± 2	0.20 ± 0.02
100-10-10	293 ± 126	391	n/a	n/a
100-10-18	308 ± 128	246	n/a	n/a
100-24-10	307 ± 127	247	n/a	n/a
100-24-18	344 ± 130	205	n/a	n/a
140-4-10	312 ± 135	251	n/a	n/a
140-8-18	413 ± 209	227	n/a	n/a
200-10-10	n/a	n/a	n/a	n/a
200-10-18	n/a	n/a	n/a	n/a
200-24-10	n/a	n/a	n/a	n/a
200-24-18	n/a	n/a	n/a	n/a

*Table S3 Statistical analysis of the particle sizes measured by SEM. One-way analysis of variance (ANOVA) was used to determine the statistical significance (at the p = 0.05 level) in the difference of means for particle size measurements. A Fisher's LSD (if sample means had unequal variance) post hoc test was used to determine which sample means differed significantly. Samples which share a group number are not statistically different. * Data from previously published work.[1]*

Sample	Mean particle size (nm)	Groups of statistical equivalence					
Previous work*	670	1					
90-8-10	206		2				
90-4-18-1	**162**			**3**			
90-4-18-2	**151**			**3**			
90-4-18-3	**159**			**3**			
90-4-18-4	**146**			**3**			
90-4-18-5	**147**			**3**			
90-4-18-6	**152**			**3**			
100-10-10	293				4		
100-10-18	308				4	5	
100-24-10	307				4	5	
100-24-18	344					5	
140-4-10	312				4	5	
140-8-18	413						6

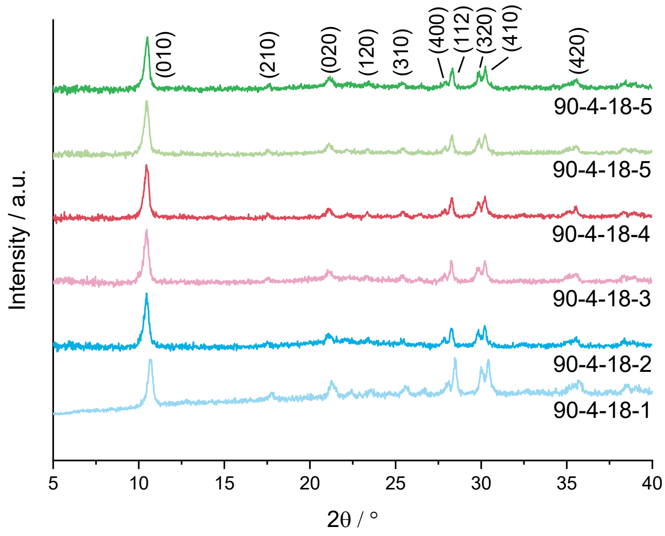

Figure S4 XRD patterns for the replicates of LTbH synthesised at the optimised conditions (90 °C, 4-hour incubation time, 18 ml fill volume). The LTbH system is indexed to the orthorhombic Pca2$_1$ space group.

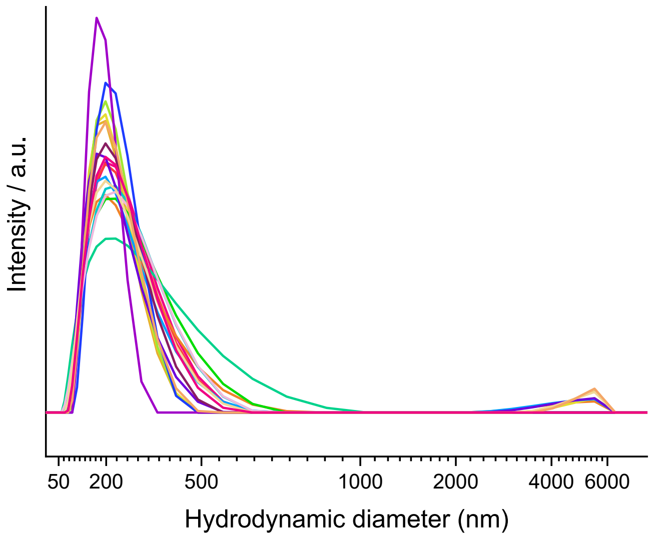

Figure S5 Superimposed hydrodynamic diameter data from DLS experiments on all size-optimised LTbH samples (each sample suspension was prepared once and measured in triplicate).

Application of optimised synthetic method to other LRHs (R = Pr, Nd, Gd, Dy, Er, Yb)

Table S4 A summary of (010) reflection positions and refined unit cell parameters of LRH materials. (R = Pr, Nd, Gd, Dy, Er, Yb). Note: LYbH was not phase pure, data shown is for most abundant phase.

Material	2θ / °	a / Å	b / Å	c / Å
**LPrH**	10.24	12.49 ± 0.02	8.60 ± 0.01	7.14 ± 0.01
**LNdH**	10.24	12.54 ± 0.02	8.70 ± 0.01	7.02 ± 0.02
**LGdH**	10.50	12.71 ± 0.02	8.42 ± 0.01	7.047 ± 0.01
**LDyH**	10.52	12.52 ± 0.02	8.37 ± 0.02	7.05 ± 0.02
**LErH**	10.58	12.60 ± 0.01	8.41 ± 0.00	7.12 ± 0.00
**LYbH**	10.58	12.56 ± 0.03	8.38 ± 0.01	7.02 ± 0.03

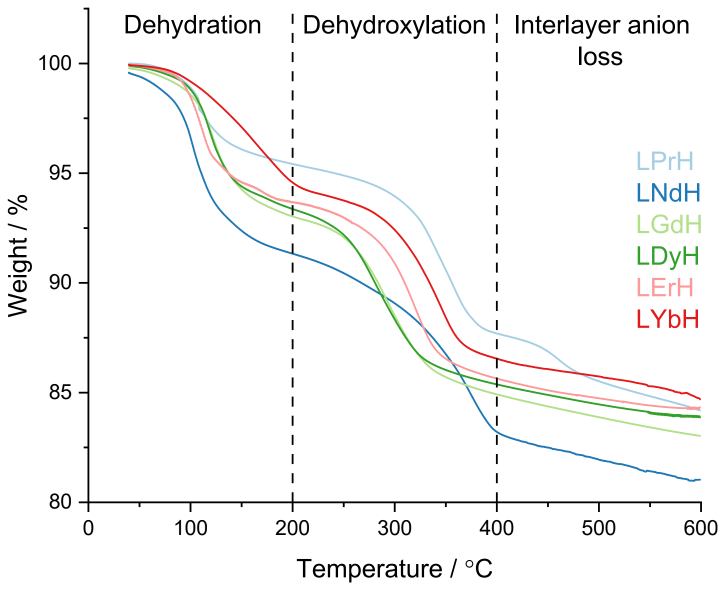

Figure S6 Thermograms of the various LRH materials (R = Pr, Nd, Gd, Dy, Er, Yb).

Table S5 Summary of observed (and calculated) values for elemental analysis and TGA data.

Material	%C	%H	Mass loss % (H_2O)
**LPrH-Cl**	1.24 (1.30)	1.53 (1.70)	4.77 (4.34)
**LNdH-Cl**	1.22 (1.24)	1.77 (1.97)	8.15 (7.24)
**LGdH-Cl**	0.42 (0.39)	1.59 (1.84)	6.83 (6.78)
**LDyH-Cl**	0.44 (0.39)	1.56 (1.53)	6.45 (5.74)
**LErH-Cl**	0.24 (0.25)	1.64 (1.68)	6.35 (5.62)
**LYbH-Cl**	0.07 (0.06)	1.63 (1.63)	6.03 (5.48)

Application of optimised synthetic method to generate mixed Gd/Tb LRH systems

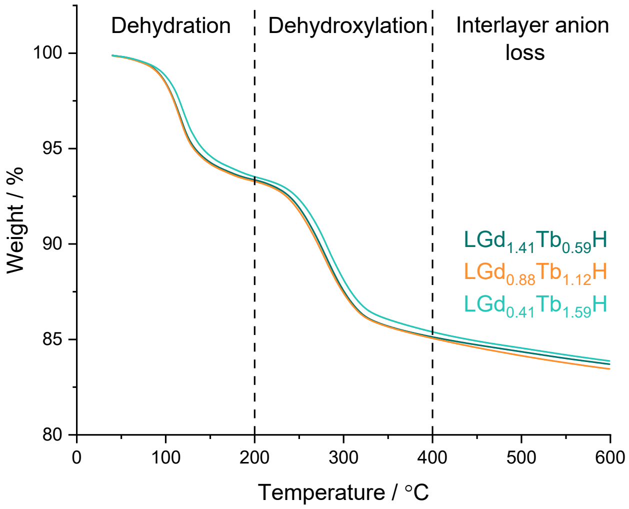

Figure S7 Thermograms of mixed LRH materials with varying composition (R = Gd/Tb).

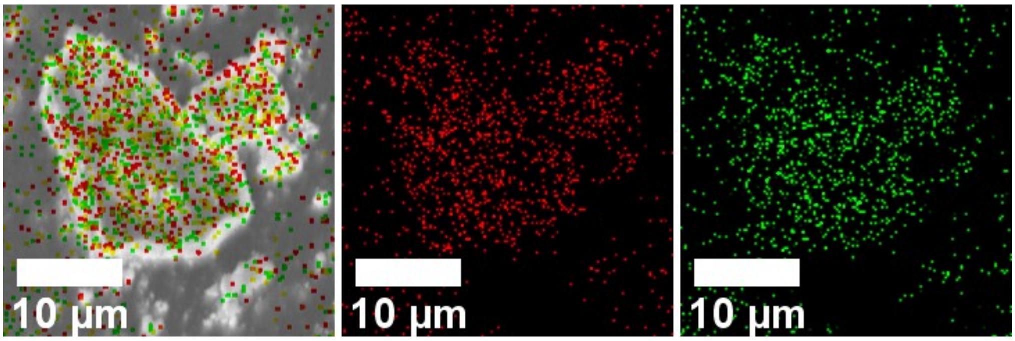

Figure S8 Representative SEM image used for EDXS analysis (sample $LGd_{1.41}Tb_{0.59}H$-Cl). Gadolinium and terbium atoms detected are shown in red and green respectively; yellow indicates sites which are either Gd or Tb.

Table S6 Summary of observed (and calculated) values for elemental analysis and TGA data.

Sample	% C	% H	Mass loss % (H_2O)
**$LGd_{1.41}Tb_{0.59}H$-Cl**	0.30 (0.28)	1.62 (1.78)	6.14 (6.22)
**$LGd_{0.88}Tb_{1.12}H$-Cl**	0.30 (0.28)	1.60 (1.78)	6.18 (6.21)
**$LGd_{0.41}Tb_{1.59}H$-Cl**	0.35 (0.32)	1.58 (1.74)	5.94 (5.83)

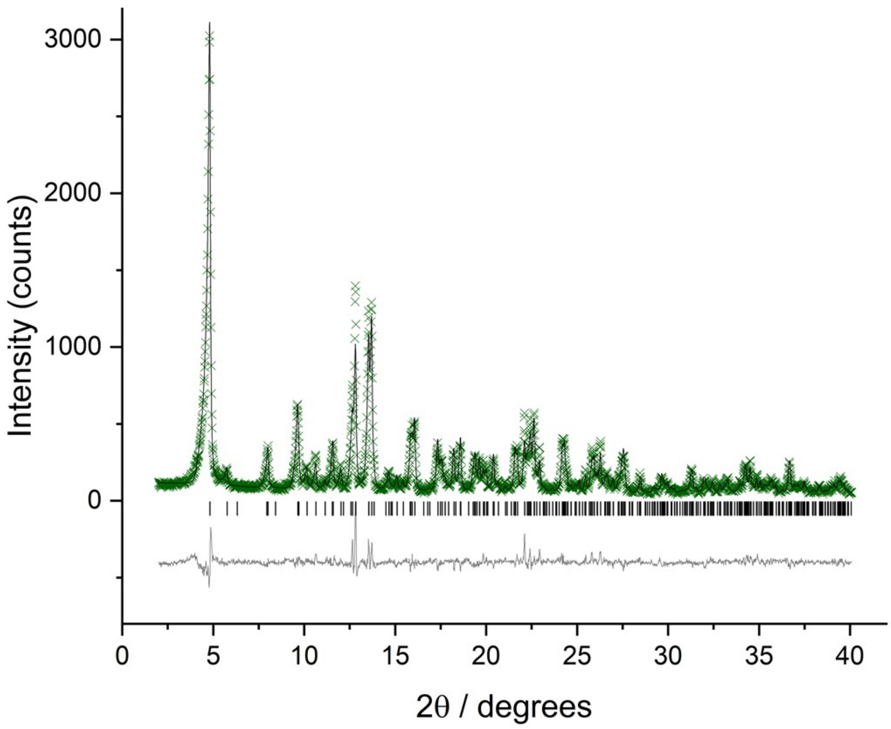

Figure S9 Observed (crosses), calculated (upper line), and difference (lower line) profiles for Rietveld refinement of LGdH-Cl. Tick marks show the positions of allowed reflections.

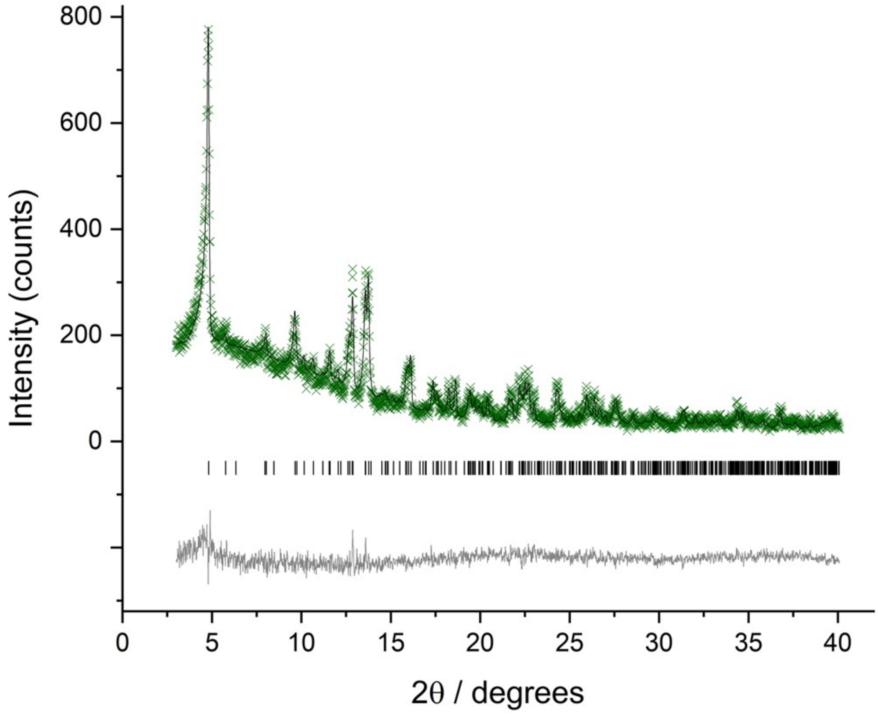

Figure S10 Observed (crosses), calculated (upper line), and difference (lower line) profiles for Rietveld refinement of LTbH-Cl. Tick marks show the positions of allowed reflections.

Table S7 Rietveld refinement details for LGdH-Cl, $LGd_{1.41}Tb_{0.59}H$-Cl, and LTbH-Cl. The initial model employed was the structure of layered ytterbium hydroxychloride, LYbH-Cl (ICSD No. 419745).[3]

	Orthorhombic, space group $Pca2_1$			
**LGdH-Cl**	2540 points, 23 parameters, χ^2 = 1.44, R(F) = 4.33 %, Rwp = 11.07 %			
	a / Å	b / Å	c / Å	V / Å^3
	12.8648(8)	8.4310(5)	7.2934(4)	791.06(6)
**$LGd_{1.41}Tb_{0.59}H$-Cl**	2540 points, 23 parameters, χ^2 = 1.44, R(F) = 5.16 %, Rwp = 10.69 %			
	a / Å	b / Å	c / Å	V / Å^3
	12.8043(7)	8.4191(4)	7.2571(3)	782.32(5)
**LTbH-Cl**	2473 points, 17 parameters, χ^2 = 1.14, R(F) = 11.95 %, Rwp = 11.95 %			
	a / Å	b / Å	c / Å	V / Å^3
	12.8150(19)	8.4288(12)	7.2625(9)	784.47(15)

$$\chi^2 = \frac{1}{N}\sum_i \frac{(y_{c,i} - y_{o,i})^2}{\sigma^2[y_{o,i}]} \qquad R(F) = \frac{\sum |F_o| - |F_c|}{\sum |F_o|} \qquad Rwp = \left[\frac{\sum_i w_i (y_{c,i} - y_{o,i})^2}{\sum_i w_i y_{o,i}^2}\right]^{1/2}$$

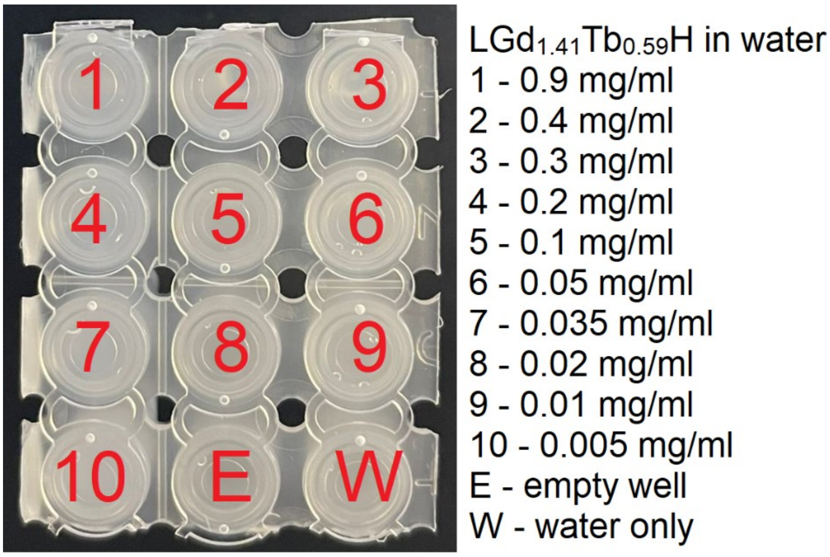

Figure S11 Layout of MR imaging phantom consisting of a dilution series of $LGd_{1.41}Tb_{0.59}H$ in water in a section of a well plate (each well is 300 µl in volume, filled with 300 µl of suspension at the indicated concentration (in mg/ml). Note: suspensions 8, 9, and 10 were excluded from analysis due to low signal change relative to water.

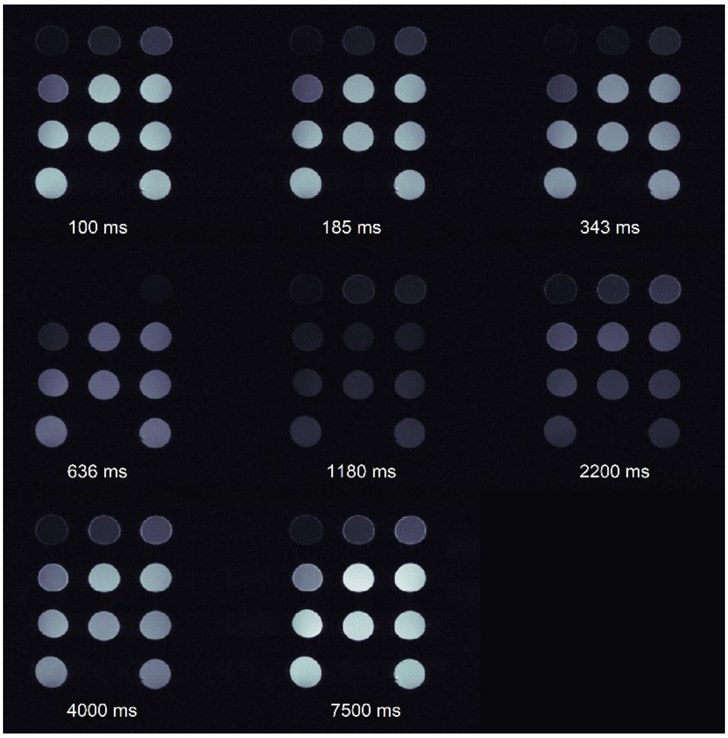

Figure S12 Representative T_1-weighted images of $LGd_{1.41}Tb_{0.59}H$ phantom (shown in Figure S11) at various inversion times.

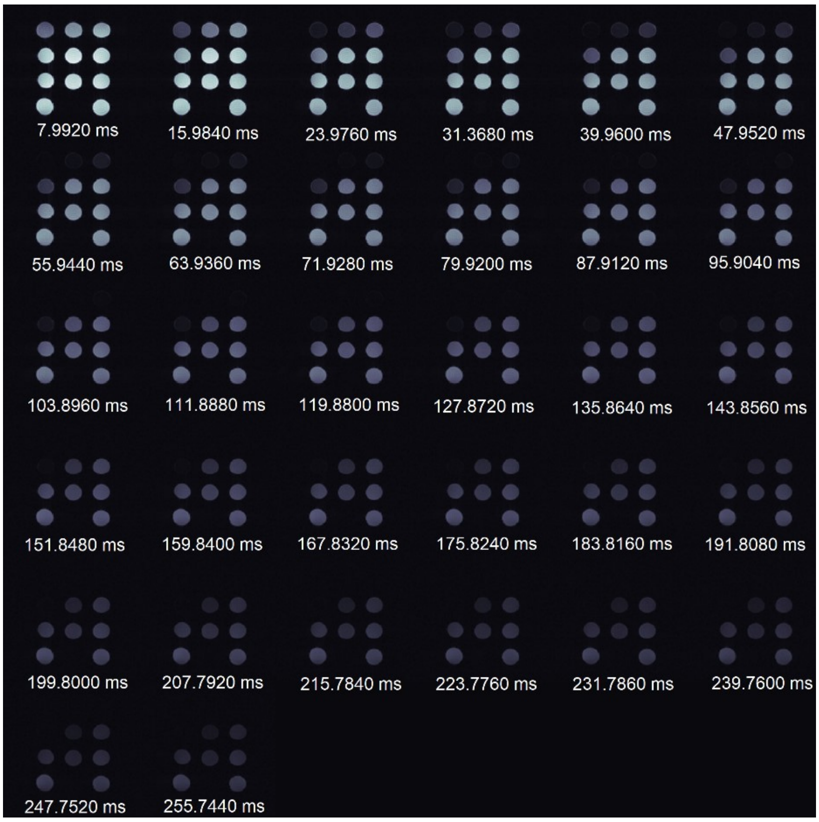

Figure S13 Representative T_2-weighted images of $LGd_{1.41}Tb_{0.59}H$ phantom (shown in Figure S11) at various echo times.

References

1.) Strimaite, M. *et al.* Layered terbium hydroxides for simultaneous drug delivery and imaging. *Dalt. Trans.* **50**, 10275–10290 (2021).

2.) Zehnder, R. *et al.* Investigation of the Structural Properties of an Extended Series of Lanthanide Bis-hydroxychlorides Ln(OH)2Cl (Ln = Nd-Lu, except Pm and Sm). *Inorg. Chem.* **49**, 4781-4790 (2010).

3.) Poudret, L., Prior, T. J., McIntyre, L. J. & Fogg, A. M. Synthesis and crystal structures of new lanthanide hydroxyhalide anion exchange materials, Ln2(OH)5X·1.5H2O (X = Cl, Br; Ln = Y, Dy, Er, Yb). *Chemistry of Materials* **20**, 7447–7453 (2008).
